# Streamflow Prediction Using Complex Networks

**DOI:** 10.3390/e26070609

**Published:** 2024-07-18

**Authors:** Abdul Wajed Farhat, B. Deepthi, Bellie Sivakumar

**Affiliations:** Department of Civil Engineering, Indian Institute of Technology Bombay, Powai, Mumbai 400 076, India; awajed13@gmail.com (A.W.F.); 204040009@iitb.ac.in (B.D.)

**Keywords:** clustering coefficient, coefficient of variation, contiguous United States, distance threshold, nearest neighbor approach, network theory

## Abstract

The reliable prediction of streamflow is crucial for various water resources, environmental, and ecosystem applications. The current study employs a complex networks-based approach for the prediction of streamflow. The approach consists of three major steps: (1) the formation of a network using streamflow time series; (2) the calculation of the clustering coefficient (CC) as a network measure; and (3) the use of a clustering coefficient-based nearest neighbor search procedure for streamflow prediction. For network construction, each timestep is considered as a node and the existence of link between any node pair is identified based on the difference (distance) between the streamflow values of the nodes. Different distance threshold values are used to identify the critical distance threshold to form the network. The complex networks-based approach is implemented for the prediction of daily streamflow at 142 stations in the contiguous United States. The prediction accuracy is quantified using three statistical measures: correlation coefficient (R), normalized root mean square error (NRMSE), and Nash–Sutcliffe efficiency (NSE). The influence of the number of neighbors on the prediction accuracy is also investigated. The results, obtained with the critical distance threshold, reveal that the clustering coefficients for the 142 stations range from 0.799 to 0.999. Overall, the prediction approach yields reasonably good results for all 142 stations, with R values ranging from 0.05 to 0.99, NRMSE values ranging from 0.1 to 12.3, and the NSE values ranging from −0.89 to 0.99. An attempt is also made to examine the relationship between prediction accuracy and the catchment characteristics/streamflow statistical properties (drainage area, mean flow, coefficient of variation of flow). The results suggest that the prediction accuracy does not have much of a relationship with the drainage area and the mean streamflow values, but with the coefficient of variation of flow. The outcomes from this study are certainly promising regarding the application of complex networks-based concepts for the prediction of streamflow (and other hydrologic) time series.

## 1. Introduction

Streamflow is an essential component of the hydrologic cycle, with important implications for our water resources, environment, and ecosystems. Streamflow arises from complex and nonlinear interactions between climate inputs and landscape characteristics that vary in both space and time. As a result, streamflow monitoring, modeling, and prediction are highly challenging.

The past century has witnessed a wide range of approaches and methods for the modeling and prediction of streamflow (and other hydrologic processes). Among these, time series analysis techniques have played a vital role in recent decades. Early linear stochastic models (e.g., autoregressive (AR), moving average (MA), autoregressive moving average (ARMA), autoregressive integrated moving average (ARIMA)) became popular in the 1960s and 1970s and were subsequently applied for streamflow modeling and prediction by numerous studies [1,2,3,4,5,6]. Advances in computer and measurement technologies since the 1970s led to the emergence of many nonlinear time series methods and their applications for streamflow modeling and prediction. These methods include artificial neural networks, support vector machines, entropy theory, wavelets, and chaos theory, among others [7,8,9,10,11]. Several studies have also proposed and/or applied hybrid/integrated models, coupling two or more different methods [12,13,14,15,16]. Such studies have used combinations of two or more methods among, for example, artificial neural networks, wavelets, support vector machines, chaos theory, deep learning algorithms, short long-term memory method, and empirical mode decomposition. A comprehensive review of the applications of data-based techniques in hydrology can be found in [17].

Recent developments in network theory (or graph theory), especially the science of complex networks [18,19], have been gaining increasing attention and applications in streamflow (and other hydrologic) studies—a network is a set of points connected by a set of lines. Complex networks-based concepts provide new avenues to unravel the nature and extent of connections in large, complex, and dynamically evolving systems. Over the past decade or so, many studies have applied the complex networks-based concepts for the analysis of streamflow time series. Such applications have mainly focused on spatial connections [20,21,22], temporal connections [23,24,25], spatio-temporal connections [26], and catchment classification [21,27,28]. Some studies have also applied the concepts for regional flood frequency analysis [29,30], the identification of the importance of individual stations in a streamflow monitoring network [31], and the examination of streamflow behavior towards determining predictability [32]. These studies have applied a variety of complex network-based concepts and associated measures, including clustering coefficient, degree centrality (and several other centrality measures), degree distribution, shortest path length, visibility graph, and community structure, among others. Some studies on streamflow have also coupled complex network concepts with chaos theory, especially for network formation [25,26]. Despite the fact that the applications of complex network-based concepts for streamflow (and other hydrologic time series) are still in a very early stage, the results reported by the studies are certainly promising.

Encouraged by the outcomes of these studies, the present study introduces the concepts of complex networks for the prediction of streamflow. As in many other time series/data-based methods, the prediction here is based only on a single-variable time series, i.e., streamflow time series. With streamflow time series, the procedure for prediction adopted in this study involves the following steps [33]: (1) the formation of a streamflow network—each timestep is considered as a node of the network and the presence of a link between any pair of nodes is identified based on the difference (distance) between the streamflow values of the nodes; (2) calculation of clustering coefficient for the individual nodes of the network; (3) identification of the nearest neighbor for the node of interest based on clustering coefficient values; (4) preliminary prediction using the nearest node and linear approximation; and (5) improved prediction using node distance. The procedure is implemented for the prediction of daily streamflow data in the contiguous United States. Daily data over a period of 10 years (1 January 2011–31 December 2020) from each of 142 streamflow stations are analyzed. The first 7 years of streamflow data (1 January 2011–31 December 2017) are used to form the network (and for the clustering coefficient calculation and nearest neighbor identification) to predict the remaining 3 years of data (1 January 2018–31 December 2020). Three statistical measures, namely correlation coefficient (R), normalized root mean square error (NRMSE), and Nash–Sutcliffe efficiency (NSE), are used to assess the prediction accuracy. The influence of the number of neighbors on the prediction accuracy is also examined. An attempt is also made to investigate the relationship between prediction accuracy and the catchment characteristics/streamflow statistical properties (drainage area, mean flow, coefficient of variation of flow).

## 2. Materials and Methods

A network is a set of points (nodes or vertices) connected by a set of lines (links or edges). There are many measures to study the properties of networks. Some of the popular and widely applied measures include the clustering coefficient, degree centrality, betweenness centrality, degree distribution, shortest path length, and community structure, among others. Each of these measures represents a key, distinct, and unique property of a network. The clustering coefficient measures the tendency of a network to form clusters, serving as an indicator of the network’s local density. Degree centrality refers to the number of connections that a node has and, hence, serves as an indicator of the central or influential node in a network. Betweenness centrality measures a node’s influence over the transfer of information between other nodes. Degree distribution, which expresses the fraction of nodes in a network with a certain number of links, serves as a measure of spread. The average shortest path length quantifies the network’s efficiency in sending information between nodes, with the shortest path being the one with the minimum number of links between any given pair of nodes *i* and *j*. Community structure identifies groups of densely connected nodes, highlighting the network’s modular organization and aiding in understanding communities.

In the present study, clustering property of the network is considered and the clustering coefficient is used as the basis to identify the nearest node(s) in the network for prediction (its evolution in time). The details regarding the calculation of the clustering coefficient are provided in the Supplementary Material (Section S1).

In this study, the complex networks-based prediction procedure proposed by Mao et al. [33] is applied for the prediction of streamflow. The procedure involves streamflow network formation, the calculation of clustering coefficient, the identification of nearest node(s) based on clustering coefficient, preliminary prediction, and improved prediction. These steps are briefly described below. The MATLAB software R2022a version is used for the analysis and plotting.

### 2.1. Network Formation

There exist several methods for the formation of a network, depending on whether the network under consideration is spatial or temporal. For a temporal network, such as the one considered in this study, the available methods include horizontal visibility algorithm [34], natural visibility algorithm [35], and distance threshold [25,36], among others. In the present study, the difference in the values between any two timesteps in the streamflow time series is considered to identify the presence of links and to form the network.

Let us consider a time series (e.g., streamflow) $Y_{t}$, where t = 1, 2, …, *N*. If each timestep of $Y_{t}$ is treated as a node in the network, then there will be *N* nodes. The existence of a link between any two nodes *i* and *j* is identified by determining the difference ($d_{ij}$) in the values between the nodes *i* and *j* (i.e., *Y_i_* and *Y_j_*) and comparing it against a distance threshold value. For instance, if the value of $d_{ij}$ between the node pair *i* and *j* is less than the assumed distance threshold, then there exists a link between them, otherwise not.

It is appropriate to note that different links can be identified for different distance threshold values, which may influence the calculation of the network measures (e.g., the clustering coefficient) and the subsequent calculations (e.g., prediction). Therefore, the identification of the optimal or critical distance threshold is important. In this study, the critical distance threshold is identified by considering different distance thresholds and determining the network density (ratio of actual links in the network to the possible number of links in the network) for each threshold. Specifically, 30 different distance threshold values between the maximum and minimum value of $d_{ij}$ among all the node pairs in the network are considered. To identify the critical threshold, the slopes of the network density values for different intervals of the distance thresholds are calculated and the distance threshold corresponding to the maximum slope value is considered as the critical threshold [25,36]. Using this critical threshold, the network is formed and the clustering coefficient value of each node in the network is computed.

### 2.2. Prediction of Streamflow Using Clustering Coefficient

With the available time series (e.g., streamflow) $Y_{t}$, where t = 1, 2, …, *N*, i.e., $Y_{1}$, $Y_{2}$,…, $Y_{N}$, the problem is to predict the value of $Y_{N+1}$. The methodology adopted in this study for predicting the value of $Y_{N+1}$ involves three steps: (1) the identification of the nearest neighbor(s) based on the node similarity (i.e., clustering coefficient) in the network; (2) preliminary prediction of time series based on the nearest neighbor(s); and (3) improvement in the preliminary prediction using the calculation of node distance. These steps are described next.

#### 2.2.1. Identification of Nearest Neighbor Based on Clustering Coefficient

Each timestep of the time series $Y_{1}$, $Y_{2}$, $Y_{3}$,…, $Y_{N}$ acts as a node in the network. The initial prediction starts with the identification of the nearest neighbor(s) for the last node *N* in the network, with value $Y_{N}$. Hereafter, for the purpose of simplicity, the nodes are also denoted as $Y_{t},\;t$ = 1, 2, …, *N*. The steps to identify the nearest neighbor(s) for the node $Y_{N}$ are as follows:

**Step 1:** Form the network using the time series, as described in Section 2.1.
**Step 2:** Calculate the clustering coefficient (CC) value of each node. Since there are N nodes (corresponding to *N* timesteps), there are N clustering coefficient values.
**Step 3:** Find the nearest neighbor of $Y_{N}$ based on the minimum values of $\left\|{CC}_{N}-{CC}_{t}\right\|$ for t<N. If the difference between the clustering coefficient value of the nodes $Y_{M}$ (i.e., (${CC}_{M})$) and $Y_{N}\;(i.e.,\left({CC}_{N}\right))$ is the minimum among all the nodes, then $Y_{M}$ will be the identified nearest neighbor for $Y_{N}$ based on the clustering coefficient.

The nearest neighbor node identified based on the clustering coefficient value is further used for the preliminary prediction of the time series.

#### 2.2.2. Preliminary Prediction

The preliminary prediction consists of mainly two steps:

**(i)**  **Step 1: Adjacent node prediction method**

In a dynamic system, the current time directly influences the immediate future time. According to this, node $Y_{N}$ has direct influence on node $Y_{N+1}$. In other words, the future node $Y_{N+1}$ is close to the current node $Y_{N}$ or any other node that has similar properties as that of $Y_{N}$. Therefore, the problem is to identify the node that has the highest similarity to node $Y_{N}$. In this study, the clustering coefficient is used as a measure to identify similar nodes. Therefore, if, for instance, the clustering coefficient of node $Y_{M}$ is similar to that of node $Y_{N}$, then node $Y_{M}$ will be identified as the nearest neighbor. Therefore, the evolution from $Y_{N}$ to $Y_{N+1}$ is assumed to be similar to the evolution from $Y_{M}$ to $Y_{M+1}$. On the basis of such a consideration, the value of $Y_{N+1}$ is calculated as:

(1) $$Y_{N+1}=\frac{Y_{M+1}-Y_{M}}{t_{M+1}-t_{M}}\left(t_{N+1}-t_{N}\right)+Y_{N}$$

where t is the value of timestep.

**(ii)**  **Step 2: Linear approximation prediction method**

In the first step above, the current and past nodes, i.e., $Y_{N}$ and $Y_{M}$, were used independently to predict the future node $Y_{N+1}$. In the second step, the nodes $Y_{M}$ and $Y_{N}$ are directly linked to predict the future node, according to:

(2) $$Y_{N+1}=\frac{Y_{N}-Y_{M}}{t_{N}-t_{M}}\left(t_{N+1}-t_{M}\right)+Y_{M}$$

#### 2.2.3. Improved Prediction Using Node Distance

The preliminary prediction of a time series value, described above, is further improved by calculating the node distance and weight coefficient. The distance between the nodes $Y_{M}$ and $Y_{N},\;$denoted by $d_{M\to N}$, is determined as:

(3) $$d_{M\to N}=t_{N}-t_{M}$$

Similarly, the distance between the nodes $Y_{M}$ and $Y_{N+1}$ and the distance between the nodes $Y_{N}$ and $Y_{N+1}$, respectively, are calculated as:

(4) $$d_{M\to N+1}=t_{N+1}-t_{M}$$

(5) $$d_{N\to N+1}=t_{N+1}-t_{N}$$

The values of these node distances are taken into account if the node identified as a neighbor (i.e., $Y_{M}$) is too far away from node $Y_{N}$ in terms of timestep (i.e., $t_{N}\gg \;t_{M}$). In such a situation, the value of $d_{M\to N}$ is large, and the similarity of nodes in the network is weakened. Therefore, in order to quantify the degree of similarity, the node distances are considered and the weight coefficient corresponding to them are given as:

(6) $$w_{1}=\frac{{\mathrm{d}}_{N\to N+1}}{{\mathrm{d}}_{M\to N+1}}$$

(7) $$w_{2}=\frac{{\mathrm{d}}_{M\to N}}{{\mathrm{d}}_{M\to N+1}}$$

where $w_{1}$ denotes the weight coefficient of the result obtained by the adjacent node prediction method (Step 1 in Section 2.2.2) and $w_{2\;}$denotes the weight coefficient of the linear approximation prediction method (Step 2 in Section 2.2.2).

Therefore, if the results of Step 1 and Step 2 in Section 2.2.2 are denoted by $y_{1}$ and $y_{2}$, the final value of $Y_{N+1}$ is calculated as:

(8) $$Y_{N+1}=w_{1}y_{1}+w_{2}y_{2}$$

**Figure A1:**
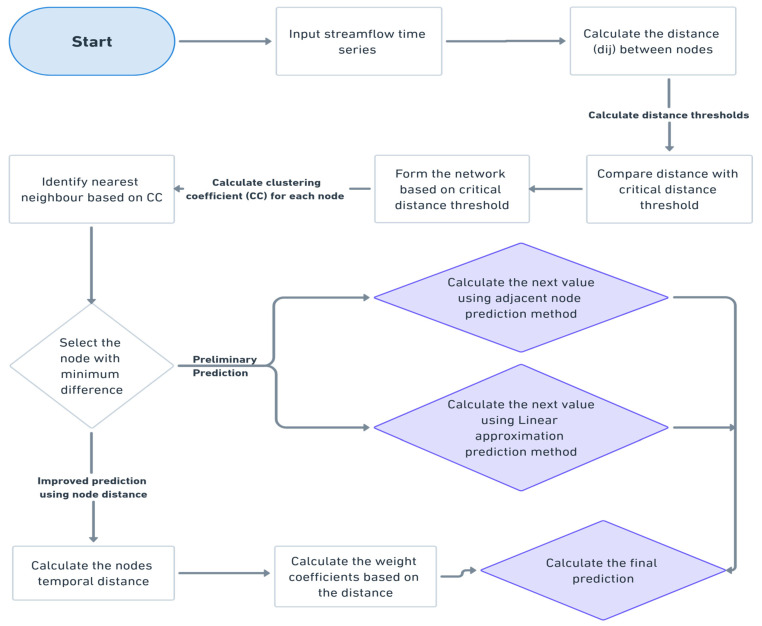
Flowchart for the complex network-based prediction approach used in this study.

A flowchart of the methodology adopted in this study is shown in Appendix A (Figure A1). The accuracy of the predicted values can be evaluated using any of the statistical evaluation measures. In this study, three evaluation measures are used: correlation coefficient (R), normalized root mean square error (NRMSE), and Nash–Sutcliffe efficiency (NSE). Each of these evaluation measures is described in Appendix B. Additionally, the effect of the number of neighbors on prediction accuracy is investigated by considering different number of neighbors. When more than one neighbor is considered, the predicted value is obtained as the average of the predicted values for all the neighbors considered.

### 2.3. Study Area and Data

**Figure 1:**
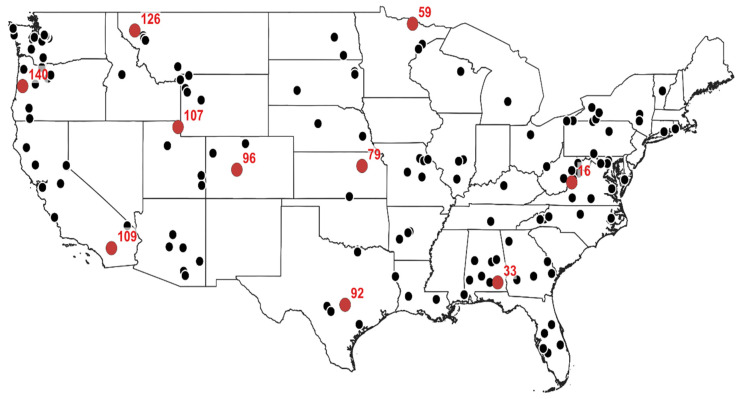
Geographical locations of the 142 streamflow gauging stations across the contiguous United States considered in this study. The ten stations marked in red are selected for the purpose of the illustration of results and discussion.

In the present study, streamflow data from the United States are used. Specifically, daily streamflow data from 142 streamflow gauging stations across the contiguous United States are analyzed. Figure 1 shows the locations of these 142 stations. The daily streamflow data are downloaded from the US Geological Survey (USGS) database (https://waterdata.usgs.gov/nwis/sw, accessed on 20 August 2021). The data used in this study are those observed over a period of 10 years, from 1 January 2011 to 31 December 2020. For the purpose of illustration of the results and discussion, ten stations are selected (marked in red in Figure 1). These stations are selected in such a way that they roughly represent the different regions across the study area (as well as the accuracy of predictions (high, medium, and low), to be discussed later).

Table 1 presents a summary of the minimum and maximum values of drainage area of the stations and some important streamflow characteristics, including the corresponding station numbers. The flow characteristics include mean, standard deviation, coefficient of variation (CV), minimum, maximum, and number of zeros. The characteristics of each of the 142 stations considered in this study are provided in Table S1 of the Supplementary Material (Section S2).

**Figure 2:**
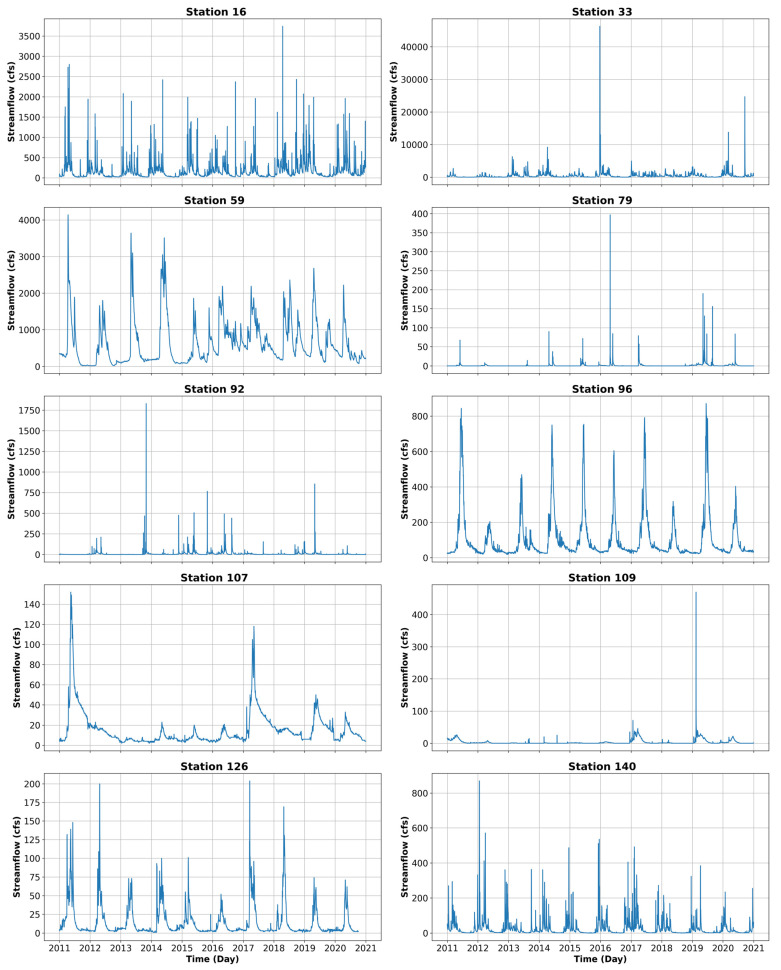
Time series plots of streamflow from ten selected stations in the United States.

Table 2 shows some basic information about the ten selected stations (see Figure 1) for illustration, including the station number, station name, state of location of the station, and drainage area of the station as well as the mean, standard deviation, and coefficient of variation of flow values. Figure 2 presents the time series plots of the flow values from these 10 stations. As seen, the streamflow time series from these 10 stations show noticeable differences in their variations and, thus, can be considered representative of the streamflow variations in the different regions across the contiguous US.

From the perspective of the variability of flow, the 10 selected stations show some noticeable differences, as can be seen from the coefficient of variation (CV) values (Table 2) and time series (Figure 2). Stations 79, 92, and 109 have relatively higher CV values (above 3.0), when compared to the others, while stations 59, 96, and 107 have relatively lower CV values, Station 59 is the only station with a CV value less than 1.0. The remaining four stations, i.e., stations 16, 33, 126, and 140, have intermediate CV values.

## 3. Results

**Figure 3:**
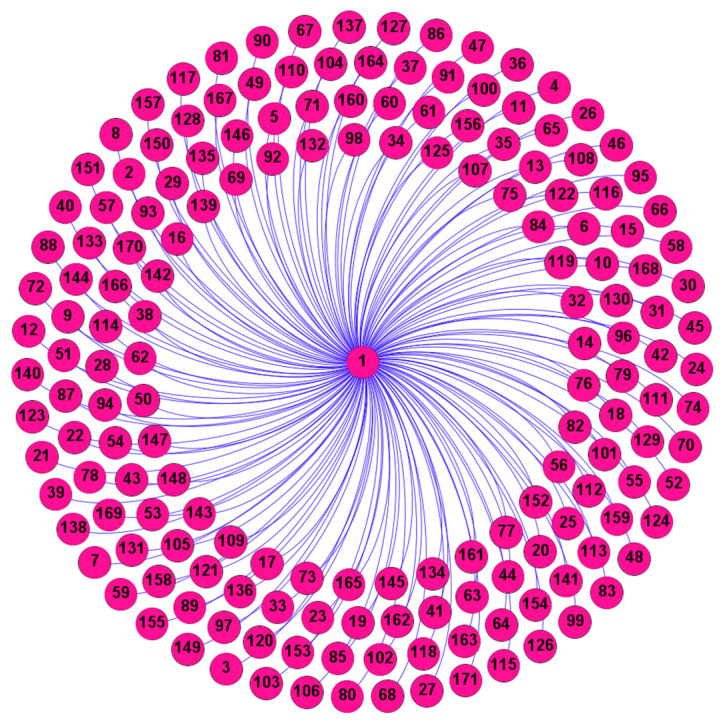
Links for the first node (day 1) in the streamflow network of Station 16 (USGS Station #2011400, Jackson River near Bacova, VA).

The prediction approach, described in Section 2, is applied for the prediction of daily streamflow in each of the 142 stations in the United States considered in this study. Each station is considered as a network, resulting in a total of 142 networks. The clustering coefficient is used as a network measure to identify the nearest neighbors in the prediction approach. To construct the network at each station, each day is treated as a node, and links between nodes are determined based on the differences in their streamflow values. The critical threshold is determined based on an analysis of 30 different threshold distances at equal intervals. After identifying the critical threshold, the network for each station is formed based on the threshold, i.e., links are identified only based on the critical threshold value. Then, the clustering coefficient of each node in the network is calculated and subsequently the average of the clustering coefficient of all the nodes in the network (i.e., the clustering coefficient of the network) is calculated. Figure 3, for instance, shows the links between node 1 (day 1) and the other nodes (days) in the network for Station 16 (USGS Station #2011400, Jackson River near Bacova, VA) based on the critical threshold determined. From this figure, it is evident that node 1 (day 1) is connected to a number of other nodes, such as node 69 (69th day), node 92 (92nd day), and node 132 (132nd day), among others. Similarly, connections for the remaining nodes (days) in the network can be identified using the same critical threshold. With the construction of the network based on such links, the prediction procedure is carried out. The first 7 years of streamflow data (January 2011–December 2017) are used to form the network, and the prediction is then performed for the remaining 3 years of data (January 2018–December 2020). Therefore, the latter period (January 2018–December 2020) may also be considered as the testing period, so to speak.

### 3.1. Clustering Coefficient

**Figure 4:**
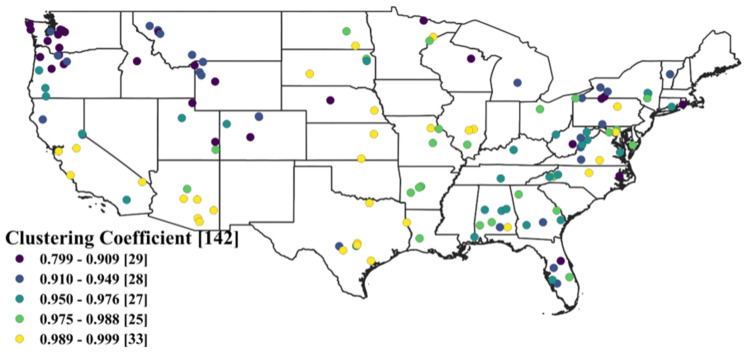
Clustering coefficient values of 142 streamflow networks across the United States. The numbers within the brackets represent the count of stations within that range of values.

Figure 4 shows the clustering coefficient (CC) values for the 142 networks (stations) considered in this study, obtained using the first seven years of streamflow data (January 2011–December 2017). The CC values range from 0.799 to 0.999, and are grouped under five categories. The numbers within the brackets represent the count of stations within that range of values. The high CC values indicate a generally strong clustering property among the nodes in each network, for the specific critical distance threshold value. Among the 142 stations, most exhibit a highly clustered property with CC values ranging from 0.910 to 0.999. The stations in the central and south-western regions are particularly prominent in this category, with values ranging from 0.989 to 0.999. Stations with CC values falling between 0.950 and 0.988 are mainly located in the eastern part of the United States, with a few stations in the western region. On the other hand, stations with lower CC values, compared to the rest, are situated in the north-western part and some in the eastern part, while stations with the lowest CC values are predominantly found in the far western part of the United States. The CC characteristics of the stations reveal that stations in close spatial proximity tend to exhibit similar network characteristics. Highly clustered stations are concentrated in geographically closer regions, whereas less-clustered stations are grouped in other geographic locations.

It is important to note that the clustering coefficient of a network varies based on the threshold used for its formation [20,37,38]. In this study, we consider the distance between values as the basis for the threshold for network formation. A higher distance leads to more links in the network, thus resulting in a higher clustering coefficient. The slope method used here to determine the critical threshold may not be the most optimal. Therefore, it is advisable to verify the results using alternative methods.

### 3.2. Streamflow Prediction

**Figure 5:**
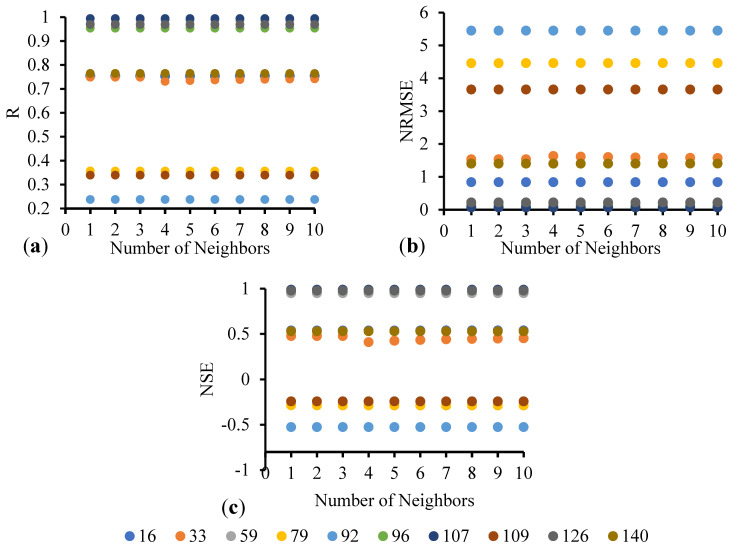
Variation in prediction accuracy with respect to the different number of neighbors for ten the selected stations: (**a**) R; (**b**) NRMSE; and (**c**) NSE.

In the neighbor search approach, different numbers of neighbors are considered in this study to examine how the number of neighbors influences the prediction accuracy. The numbers of nearest neighbors considered are from 1 to 10. Figure 5 presents the R, NRMSE, and NSE values obtained for the 10 selected stations, shown in Figure 1, for different numbers of neighbors for the prediction (or testing) period (1 January 2018–31 December 2020). As indicated in the figure, the 10 different colors represent the 10 different stations selected here for the purpose of illustration. From Figure 5a, it is evident that Station 92 (light blue color) has the lowest correlation coefficient for prediction among all the stations. For this station, even with an increased number of neighbors during prediction, there is only a very minimal increase in the value of R. Similarly, the NRMSE (Figure 5b) and NSE (Figure 5c) values do not significantly improve with an increased number of neighbors for Station 92 during prediction. Furthermore, examining stations with high accuracies (high R, low NRMSE, and high NSE), particularly Stations 59 and 126, it is observed that increasing the number of neighbors during prediction does not significantly enhance the prediction accuracy. Therefore, for stations that yield very high and very low prediction accuracy, increasing the number of neighbors has no substantial impact on the prediction accuracy. Indeed, the number of neighbors does not seem to be have any significant influence on the prediction accuracy for any station considered in this study.

**Figure 6:**
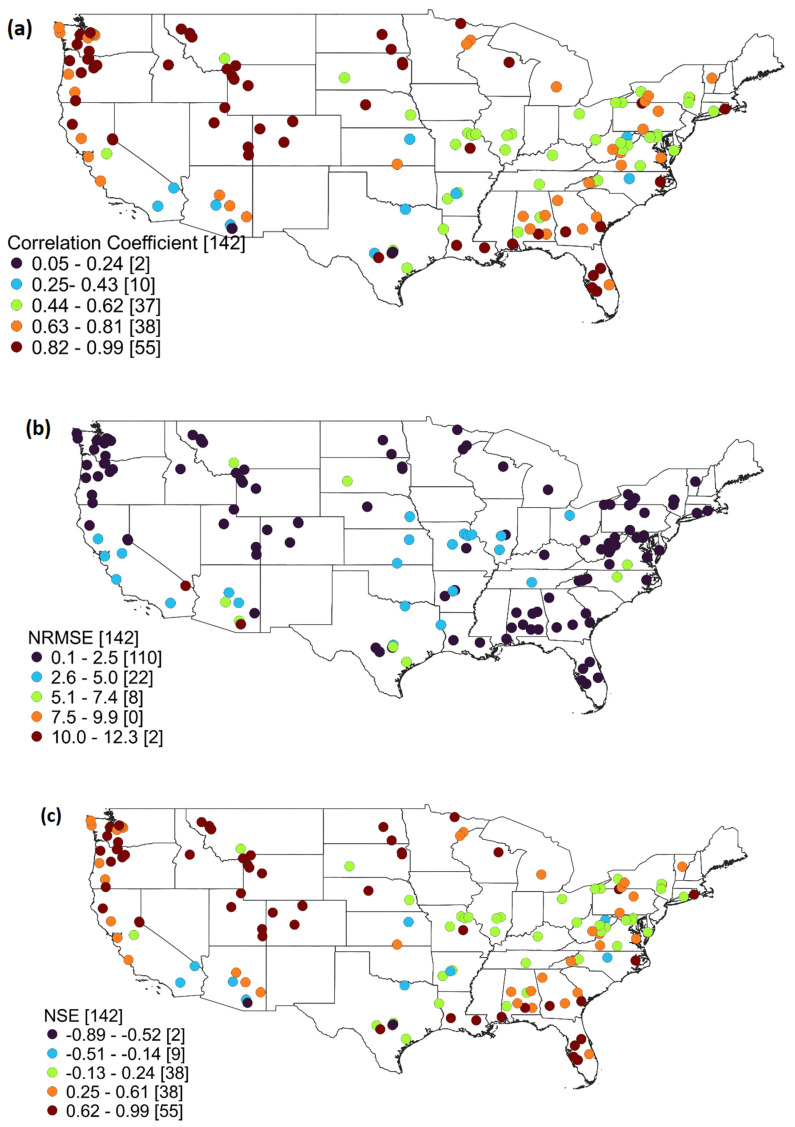
(**a**–**c**) Values of R, NRMSE, and NSE obtained for 142 stations across the United States. The numbers within the brackets represent the count of stations within that range of values.

Since there is no significant change in the prediction accuracy with respect to the number of neighbors: prediction results obtained with only one nearest neighbor are presented here for a further detailed discussion. Figure 6 presents the R, NRMSE, and NSE values obtained for all the 142 stations during the testing period (1 January 2018–31 December 2020), when only one nearest neighbor is used for prediction. For each of the three evaluation measures, the values are categorized into five ranges, as appropriate. Among the 142 stations, 55 stations exhibit an R value ranging from 0.82 to 0.99, while 38 stations have an R value between 0.63 and 0.81 (Figure 6a). The rest of the stations display R values below 0.63, with only two stations falling in the range of 0.05–0.24. Stations in the north and northwestern regions demonstrate a high level of prediction accuracy and are generally more predictable than those in the south and northeast. Considering the NRMSE values (Figure 6b), as many as 110 stations have values in the range of 0.1–2.5, with only a few stations having NRMSE values exceeding 2.5. The NSE values, which can better serve as an indicator of the accuracy of the prediction model, indicate that a total of 55 stations have a very high level of accuracy in prediction (values between 0.62 and 0.99), while 38 stations have NSE values between 0.25 and 0.61, indicating a reasonably good level of accuracy in prediction.

## 4. Discussion

**Figure 7:**
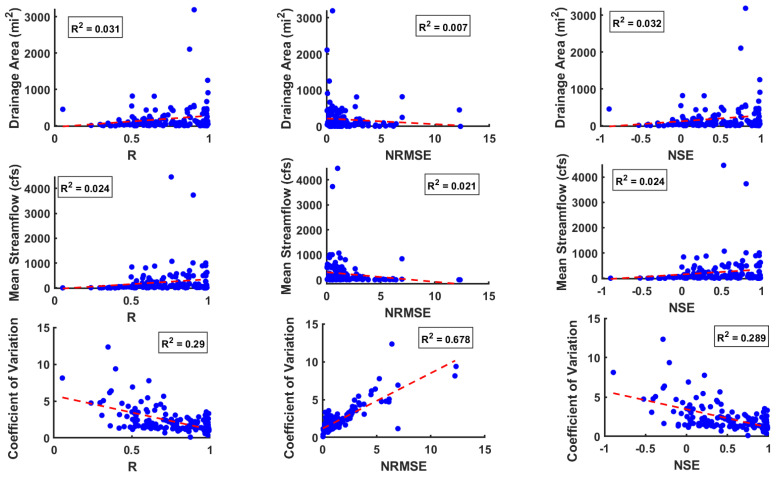
Scatter plot showing the relationship between prediction accuracies and both the statistical characteristics of streamflow data and its catchment characteristics.

As Figure 6 shows, the prediction accuracy for daily streamflow significantly varies among the 142 stations, indicating the possible presence of various influencing factors for different regions/stations. Therefore, it may be useful to examine the relationship between the prediction accuracy against the catchment characteristics and statistical properties of the streamflow data. To this end, Figure 7 presents the R, NRMSE, and NSE values against the drainage area (first row), mean of the streamflow values (second row), and coefficient of variation of the streamflow values (third row). The coefficient of determination ($R^{2}$) value between each of the three prediction accuracy measures and each of the catchment/flow statistical characteristics is also displayed in Figure 7.

The scatter plots between prediction accuracies (R, NRMSE, and NSE) and drainage area (first row) suggest that the drainage area has no significant influence on the prediction accuracy. For stations with high mean values, prediction accuracy tends to be good (second row). The results for stations with low mean flow values are somewhat mixed. While some stations with low mean values also exhibit a good prediction accuracy, others with low mean values show poor prediction accuracy. The prediction results also show that an increase in the coefficient of variation corresponds to a decrease in the prediction accuracy in terms of high R, low NRMSE, and high NSE (third row), as normally expected. In addition to this, regression analysis is also performed to quantify the relationship between the prediction accuracy (R, NRMSE, and NSE) and catchment characteristics/statistical properties of the data; see Supplementary Material (Section S3) for the results. The results from the regression analysis also suggests that coefficient of variation has significant influence on the prediction accuracy, while the drainage area and mean flow does not significantly impact the prediction accuracy metrics.

**Figure 8:**
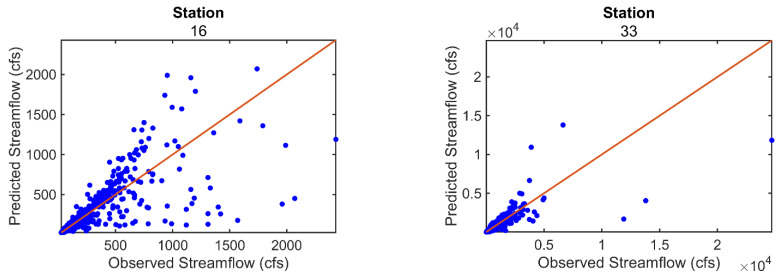

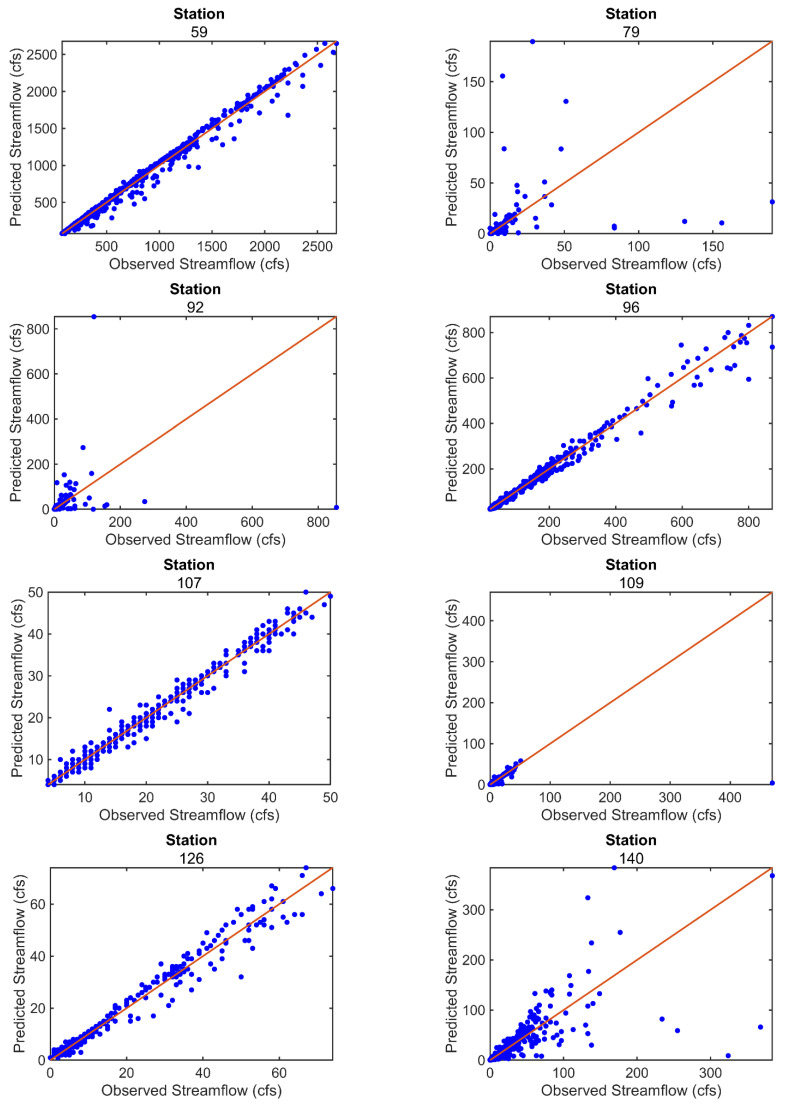
Scatter plot between observed and predicted streamflow for 10 selected stations.

Figure 8 displays the scatter plots between the observed and predicted streamflow values for the 10 selected stations (shown in Figure 1). The scatter plots show that there is generally good agreement between the observed and predicted streamflow for all 10 stations. Similar results are also obtained for most of the 142 stations, especially those stations with high prediction accuracy.

The values of R, NRMSE, and NSE obtained for these 10 stations are presented in Table 3. Among these 10 stations, stations 59, 96, 107, and 126 exhibit high NSE values (0.99, 0.98, 0.99, and 0.98, respectively), while stations 79, 92, and 109 have negative NSE values. Upon observing the time series plots of these stations in Figure 2, it is evident that the stations with negative NSE values are entirely different from the time series plots of stations with high NSE values. Some seemingly ‘outlier’ values are present in the streamflow time series of stations with negative NSE values, which may result in low NSE values for these stations.

## 5. Conclusions

In this study, a complex networks-based local approximation approach was used to predict the daily streamflow at 142 stations across the United States. Each station was considered a network, and each timestep (day) was regarded as a node in the network. A key network measure, namely clustering coefficient, was used to identify the nearest neighbors for local approximation. The prediction accuracy of the model was measured using three different metrics: correlation coefficient (R), normalized root mean square error (NRMSE), and Nash–Sutcliffe efficiency (NSE). The influence of different numbers of neighbors on the prediction accuracy was also investigated. The influence of the catchment drainage area and key statistical characteristics of the flow data on the prediction accuracy was also examined.

Considering all the 142 stations, the R values were found to range from 0.05 to 0.99, the NRMSE values from 0.1 to 12.3, and the NSE values from −0.89 to 0.99. These prediction results, with high accuracy for most of the stations, are certainly encouraging. A strong inverse relationship between the coefficient of variation of flow and the prediction accuracy was observed, as the prediction accuracy was found to decrease with an increase in the coefficient of variation, as would normally be expected. However, the drainage area did not seem to show any significant influence on the prediction accuracy.

Although the outcomes from the complex networks-based local approximation prediction approach, with the clustering coefficient as a network measure, are promising, predictions of streamflow data at some of the stations were found to be poor. One possible way to improve the predictions may be by reconstructing the data in higher dimensions, rather than the single-dimensional approach used in this study. To this end, complex network-based concepts can be combined with chaos theory concepts, such as phase-space reconstruction, to form the network [25,26]. Phase-space reconstruction-based local approximation approaches have been found to yield very good predictions for streamflow (and other) catchment-related time series [9]. On the other hand, in the present study, only the clustering coefficient was considered as the network measure to identify the nearest neighbors. However, other network measures, such as shortest path length and centrality-based measures (degree centrality, closeness centrality, and betweenness centrality), may also be useful to better identify the nearest neighbors and improve the prediction accuracy. We will explore these aspects in our future research.

## Figures and Tables

**Table 1:** Characteristics of 142 streamflow stations and daily flow data in the United States.

	Minimum	Maximum	Station
Drainage area (mi^2^)	0.84	3180	Minimum: #9423350 Maximum: #6934000
Flow mean (cfs)	0.02	4461.6	Minimum: #9423350 Maximum: #12040500
Flow standard deviation (cfs)	0.15	8267.78	Minimum: #9423350 Maximum: #6934000
Flow CV	0.11	12.34	Minimum: #6775500 Maximum: #9512280
Minimum flow (cfs)	0	369	Minimum: 63 stations Maximum: #6934000
Maximum flow (cfs)	20	178,000	Minimum: #9423350 Maximum: #6934000
Number of zeros	0	3604	Minimum: 96 stations Maximum: #9423350

**Table 2:** Some basic information and streamflow characteristics of 10 selected streamflow stations in the United States (out of the 142 stations considered in this study), marked red in Figure 1.

Station S. No.	Station No.	Station Name	State *	Drainage Area (mi^2^)	Mean (cfs)	Standard Deviation (cfs)	CV
16	2011400	Jackson river near Bacova, VA	VA	157	183.94	259.63	1.41
33	2363000	Pea river near Ariton AL	AL	498	521.77	1315.82	2.52
59	5129115	Vermilion River near Crane Lake, MN	MN	905	627.30	614.29	0.98
79	6879650	Kings C NR Manhattan, KS	KS	4.44	1.47	9.38	6.39
92	8158810	Bear Ck bl FM 1826 nr Driftwood, TX	TX	12.2	9.41	44.54	4.73
96	9107000	Taylor river at Taylor Park, CO	CO	128	102.70	135.82	1.32
107	10023000	Big Creek near Randolph, UT	UT	52.4	15.41	17.53	1.14
109	10258000	Tahquitz C NR Palm Springs CA	CA	16.9	3.42	10.47	3.06
126	12374250	Mill Cr ab Bassoo Cr nr Niarada MT	MT	19.6	14.37	21.75	1.51
140	14306340	East fork lobster creek near Alsea, OR	OR	5.7	26.85	52.69	1.96

* VA—Virginia; AL—Alabama; MN—Minnesota; KS—Kansas; TX—Texas; CO—Colorado; UT—Utah; CA—California; MT—Montana; OR—Oregon.

**Table 3:** Values of R, NRMSE, and NSE obtained during the testing period (1 January 2018–31 December 2020) for the 10 selected stations.

St. Sr. No.	Station No.	R	NRMSE	NSE
16	2011400	0.72	0.97	0.44
33	2363000	0.75	1.42	0.51
59	5129115	0.99	0.09	0.99
79	6879650	0.37	4.88	−0.27
92	8158810	0.24	5.45	−0.423
96	9107000	0.99	0.18	0.98
107	10023000	0.99	0.07	0.99
109	10258000	0.31	3.86	−0.42
126	12374250	0.99	0.22	0.98
140	14306340	0.79	1.22	0.57

## Data Availability

The streamflow data used in this study were obtained from the US Geological Survey (USGS) database (https://waterdata.usgs.gov/nwis/sw, accessed on 20 August 2021). The data may be obtained from the authors upon request.

## Supplementary Materials

The following supporting information can be downloaded at: https://www.mdpi.com/article/10.3390/e26070609/s1.

## Appendix B. Prediction Evaluation Measures

In this study, three prediction evaluation measures are used: correlation coefficient, root mean square error, and Nash–Sutcliffe efficiency.

The correlation coefficient (R) assesses the strength and direction of the linear relationship between the predicted and actual values. The normalized root mean square error (NRMSE) is a statistical metric used to assess the accuracy of a predictive model. It normalizes the root mean square error (RMSE), which measures the differences between predicted values by a model and the actual observed value. The NRMSE is obtained by normalizing the RMSE by the mean of the observed data. The Nash–Sutcliffe efficiency evaluates how well the observed data are replicated by the model predictions, indicating the predictive power of the model. The R, NRMSE, and NSE are calculated as follows:

(A1) $$R=\frac{\sum_{i=1}^{x}{(a}_{i}-a_{av})(m_{i}-m_{av})}{(x-1)S_{a}S_{m}}$$

(A2) $$\mathrm{NRMSE}=\frac{\sqrt{\left(\frac{1}{x}\right)\sum_{i=1}^{x}{(a}_{i}-m_{i}{)}^{2}}}{a_{av}}$$

(A3) $$\mathrm{NSE}=1-\frac{\sum_{i=1}^{x}(a_{i}-m_{i}{)}^{2}}{\sum_{i=1}^{x}(a_{i}-a_{av}{)}^{2}}$$

where $a_{i}$ and $m_{i}$ are observed and predicted values, $a_{av}$ and $m_{av}$ are the means of observed and predicted values, $s_{a}$ and $s_{m}$ are the standard deviations of observed and predicted values, and x is the number of observations.
