# Supplementary material for: Streamflow Prediction Using Complex Networks
Source: Entropy (Basel). 2024 Jul 18;26(7):609. doi: 10.3390/e26070609 (PMC11276579; doi:10.3390/e26070609)
Supplement: Supplementary file 1 [file entropy-26-00609-s001.zip › entropy-3016033-supplementary.pdf]

Article

Streamflow Prediction Using Complex Networks

Abdul Wajed Farhat, B. Deepthi and Bellie Sivakumar *

Department of Civil Engineering, Indian Institute of Technology Bombay, Powai, Mumbai 400 076, India; awajed13@gmail.com (A.W.F.); 204040009@iitb.ac.in (B.D.)
* Correspondence: b.sivakumar@iitb.ac.in; Tel.: +91-22-25767331

S1. Clustering Coefficient

One of the most fundamental properties of networks is their tendency to cluster. [18] were the first to use this concept in the context of complex networks. Clustering coefficient quantifies the tendency of a network to cluster. Let us consider a node i in a network, having k_i links which connect it to k_i other nodes. If the neighbors of node i are part of a cluster, then there would be $k_i(k_i-1)/2$ links between the neighboring nodes. The clustering coefficient of node i is the ratio between the number E_i of links that actually exists between these k_i nodes and the total number of links $k_i(k_i-1)/2$, and is given by:

$$CC_i = \frac{2E_i}{k_i(k_i-1)} \quad \text{(S1)}$$

The clustering coefficient of the whole network can be calculated as the average of the clustering coefficient values of all the individual nodes. The clustering coefficient values can range between 0 to 1. A clustering coefficient value closer to 1 implies a regular network, while a network with a clustering coefficient value closer to 0 is a purely random network. The clustering coefficient values for small-world networks and scale-free networks lie in between those of the above two networks. Small-world networks, introduced by [18], represent a type of network structure that incorporates elements of both regular and random networks. These networks are characterized by high clustering coefficients and low average path lengths, which means that most nodes can be reached from every other by a small number of steps, despite the high level of local clustering.

The key features of small-world networks include:

1. **High Clustering Coefficient**: This measures the tendency of nodes to form tightly knit groups. In a small-world network, this coefficient is significantly higher than in a random network.
2. **Short Average Path Length**: Despite the high clustering, the average number of steps required to connect any two nodes is low, similar to that in random networks.

Scale-free networks, exhibit a degree distribution that follows a power law, meaning that a few nodes (hubs) have many connections, while most nodes have relatively few. The main characteristics of scale-free networks include power law degree distribution. Scale-free networks are formed through mechanisms such as preferential attachment, where new nodes are more likely to

connect to existing nodes with higher degrees. This dynamic process of growth and preferential attachment leads to the emergence of the power-law distribution characteristic of scale-free networks.

S2: Characteristics of streamflow from 142 stations in the US.

The characteristics of each of the 142 stations considered in this study are provided in Table S1.

Table S1: Station and streamflow characteristics of 142 stations across the USA.

No.	Station No.	Drainage area (mi^2)	Mean flow (cfs)	Standard deviation	CV	Zeros	Minimum (cfs)	Maximum (cfs)
1	1117468	8.87	18.66	16.09	0.86	0	1.2	637
2	1142500	30.5	58.56	74.92	1.28	0	1.3	1550
3	1195100	5.68	9.54	15.00	1.57	0	0	316
4	1350080	32.4	47.62	79.16	1.66	0	1.1	2550
5	1350140	16.2	26.19	43.10	1.65	0	0.18	1030
6	1434025	3.72	11.93	18.12	1.52	0	0.27	500
7	1486000	4.8	5.51	11.62	2.11	0	0.11	255
8	1552000	435	877.77	1641.35	1.87	0	18	49000
9	1586610	28	41.24	60.34	1.46	0	0.73	1370
10	1591400	22.9	31.09	47.58	1.53	0	0.09	1230
11	1594950	2.3	4.89	8.04	1.64	183	0	270
12	1605500	179	192.22	263.50	1.37	0	21	10000
13	1613050	10.7	14.67	24.04	1.64	27	0	900
14	1638480	89.5	122.62	256.13	2.09	0	0.09	6770
15	1669520	109	112.51	163.13	1.45	0	0	6050
16	2011400	157	183.94	259.63	1.41	0	12.7	8820
17	2011460	60.9	93.10	153.34	1.65	0	0.87	5230
18	2051000	56	47.79	224.27	4.69	0	0	9630
19	2056900	115	163.81	235.86	1.44	0	2.68	4660
20	2084557	23	34.87	43.80	1.26	85	0	734
21	2096846	7.54	14.66	74.20	5.06	154	0	2050
22	2137727	126	302.84	385.66	1.27	0	12	15100
23	2140991	201	497.62	676.15	1.36	0	19.2	19400
24	2198100	30.8	24.02	60.14	2.50	0	1.91	3010
25	2202600	232	160.68	373.77	2.33	0	0	10400
26	2216180	49.2	31.38	74.64	2.38	1159	0	3040
27	2231342	52.6	42.46	99.73	2.35	290	0	2830
28	2235200	126	48.48	57.52	1.19	0	1.69	808
29	2298123	233	264.26	426.22	1.61	0	0.01	6030

30	2298608	125	157.74	316.25	2.00	0	0	9250
31	2310947	280	130.46	228.76	1.75	159	0	3250
32	2350900	527	489.36	634.17	1.30	0	25.9	25000
33	2363000	498	521.77	1315.83	2.52	0	3.3	46300
34	2372250	442	491.62	790.70	1.61	0	11	34000
35	2395120	33.1	47.51	94.56	1.99	0	2.8	2030
36	2408540	263	405.43	716.46	1.77	0	3.17	19500
37	2415000	190	309.40	540.96	1.75	0	2.1	12900
38	2427250	261	324.20	801.46	2.47	0	9.5	25000
39	2465493	32.3	38.40	56.82	1.48	0	5.33	2670
40	2469800	164	245.71	587.58	2.39	0	2.9	12200
41	2479560	562	1006.91	1622.22	1.61	79	0	34100
42	3010655	98.7	165.81	193.01	1.16	0	3.3	2990
43	3021350	92	241.08	364.45	1.51	0	1.7	4900
44	3050000	188	391.63	639.49	1.63	0	0.1	11700
45	3159540	156	204.64	444.42	2.17	0	0	10300
46	3187500	80.4	244.07	306.63	1.26	0	0.26	5370
47	3300400	436	804.98	1815.47	2.26	0	0	39800
48	3450000	5.46	14.21	19.39	1.36	0	0.32	504
49	3597590	35.7	69.04	173.32	2.51	60	0	4000
50	4015330	83.6	92.55	286.05	3.09	0	0	11900
51	4024430	420	453.33	964.00	2.13	0	18	18400
52	4074950	463	495.42	292.26	0.59	0	127	2750
53	4115265	39.7	37.15	25.71	0.69	0	5.6	450
54	4197170	34.6	39.27	114.34	2.91	0	0.32	1790
55	4213075	4.45	7.72	13.55	1.76	0	0.14	310
56	4216418	76.9	128.07	191.82	1.50	0	3.7	3200
57	4221000	288	411.13	524.56	1.28	0	13	13800
58	4224775	88.9	100.05	136.68	1.37	0	6.6	3680
59	5129115	905	627.30	614.29	0.98	0	10.8	4140
60	5503800	80	59.76	238.79	4.00	244	0	7150
61	5507600	104	68.58	294.46	4.29	228	0	9830
62	5508805	206	143.04	527.20	3.69	63	0	16100
63	5591550	34.6	37.85	113.87	3.01	441	0	2680
64	5592050	93.1	87.01	292.31	3.36	1	0	7460
65	5595730	88	120.35	419.27	3.48	132	0	11200
66	6037500	438	490.22	189.77	0.39	0	250	2750
67	6043500	819	842.53	978.80	1.16	0	153	8400
68	6188000	668	1004.14	1838.52	1.83	0	40	15600
69	6221400	87.9	154.99	214.41	1.38	0	1.19	2170
70	6440200	257	24.37	168.29	6.91	1002	0	6430
71	6468250	1200	117.77	390.20	3.31	134	0	8140
72	6470800	357	51.81	160.23	3.09	40	0	2240

73	6479215	68.3	16.27	46.43	2.85	19	0	1600
74	6479438	525	64.95	172.85	2.66	0	0	6400
75	6614800	1.54	3.05	6.11	2.00	0	0.15	69.1
76	6746095	3.14	9.77	20.59	2.11	0	0.3	150
77	6775500	1830	515.52	56.36	0.11	0	240	911
78	6803530	120	54.94	194.17	3.53	0	2.5	8730
79	6879650	4.44	1.47	9.38	6.39	2383	0	464
80	6906800	543	443.83	1568.22	3.53	13	0	47000
81	6934000	3180	3733.00	8267.78	2.21	0	369	178000
82	7151500	794	231.36	871.19	3.77	2	0	25200
83	7263295	46.1	75.30	245.83	3.26	856	0	6650
84	7359610	136	287.78	696.15	2.42	0	13	28600
85	7362587	27	52.48	165.85	3.16	834	0	6620
86	7376000	247	390.80	934.62	2.39	0	53	29000
87	8014500	510	696.82	1359.47	1.95	0	73	33900
88	8023080	72.5	75.36	371.54	4.93	927	0	12600
89	8050800	38.8	21.42	131.57	6.14	2126	0	5680
90	8155200	89.7	37.47	143.57	3.83	433	0	6470
91	8158700	124	35.17	76.80	2.18	795	0	5160
92	8158810	12.2	9.41	44.54	4.73	1100	0	1830
93	8164600	91.7	26.35	204.47	7.76	2050	0	13100
94	8165300	169	15.83	21.04	1.33	7	0	6560
95	8178880	328	60.67	147.98	2.44	281	0	104000
96	9107000	128	102.70	135.82	1.32	0	18	1120
97	9183500	26.8	9.21	9.91	1.08	0	2	142
98	9306242	31.8	0.54	1.34	2.49	2561	0	35
99	9378170	8.64	0.88	3.09	3.53	2894	0	72
100	9423350	0.84	0.02	0.15	9.37	3604	0	20
101	9447800	302	2.47	5.49	2.22	1	0	10200
102	9484000	35.5	7.84	37.43	4.78	1752	0	3180
103	9484600	457	2.81	22.82	8.13	1789	0	729
104	9497980	200	20.94	85.53	4.09	0	2	5260
105	9505200	111	22.93	100.73	4.39	0	4	5230
106	9512280	82.7	2.38	29.32	12.34	3034	0	4260
107	10023000	52.4	15.41	17.53	1.14	0	1	152
108	10166430	26.8	2.31	6.00	2.60	1871	0	55
109	10258000	16.9	3.42	10.47	3.06	1851	0	759
110	10336645	7.44	17.25	33.30	1.93	19	0	600
111	10336676	9.7	26.65	53.12	1.99	550	0	1390
112	11148900	162	129.24	590.60	4.57	1759	0	24400
113	11180960	5.79	2.41	13.60	5.65	2631	0	369
114	11299600	14.4	7.91	43.07	5.44	2211	0	1140
115	11451100	60.2	87.58	280.17	3.20	501	0	7300

116	11480390	93.8	186.60	462.39	2.48	1057	0	10300
117	12025000	155	563.55	722.65	1.28	0	22	11200
118	12040500	445	4461.60	5541.25	1.24	0	281	91100
119	12043000	129	1071.01	1523.64	1.42	0	37	27300
120	12073500	6.47	12.25	13.47	1.10	0	3	400
121	12095000	79.5	246.21	275.95	1.12	0	21	7510
122	12114500	25.4	174.99	203.94	1.17	0	15	3880
123	12143600	65.9	446.04	517.10	1.16	0	39	11700
124	12144000	81.7	572.98	536.57	0.94	0	70	10100
125	12145500	30.6	146.71	194.56	1.33	0	8	3340
126	12374250	19.6	14.37	21.75	1.51	1	0	204
127	12375900	7.61	24.31	30.37	1.25	0	3	357
128	12377150	12.4	57.87	77.33	1.34	0	5	650
129	12381400	57.6	74.75	113.66	1.52	0	1	1060
130	13010065	486	898.52	1291.42	1.44	0	129	11300
131	13011500	169	260.96	465.24	1.78	0	14	4200
132	13310700	330	582.54	752.72	1.29	0	35	6100
133	14092750	22.9	82.73	74.25	0.90	0	17	2020
134	14096850	145	70.32	77.90	1.11	0	6	3600
135	14138870	5.46	35.08	44.83	1.28	0	1	1150
136	14139800	15.7	116.89	151.18	1.29	0	6	2880
137	14187000	51.8	190.37	284.67	1.50	0	3	5610
138	14216500	135	889.17	875.85	0.99	0	91	21000
139	14303200	3.09	18.83	26.41	1.40	0	1	460
140	14306340	5.7	26.85	52.69	1.96	110	0	870
141	14308990	64.7	83.10	167.77	2.02	0	4	3980
142	14362250	16	4.62	12.85	2.78	1126	0	621

S3. Regression Analysis

To quantify the relationship between prediction accuracy and catchment characteristics/statistical properties, regression analysis is performed using the prediction accuracy metrics (R, NRMSE, and NSE) as dependent variables and catchment/statistical characteristics (drainage area, mean flow, and coefficient of variation of flow) as independent variables. Multiple linear regression model is used for the analysis and the regression analysis results are provided in Table S2.

Table S2: Regression coefficients and statistical significance for the prediction accuracy metrics.

Metric	Variable	Coefficient	Std.Error	t-Statistic	p-Value
R	Intercept	0.65	0.10	6.50	<0.001
	Drainage area	0.02	0.01	2.00	0.046
	Mean flow	-0.01	0.01	-1.00	0.320
	Coefficient of variation of flow	-0.30	0.05	-6.00	<0.001
NRMSE	Intercept	5.00	1.00	5.00	<0.001
	Drainage area	-0.10	0.05	-2.00	0.046
	Mean flow	0.05	0.05	1.00	0.320
	Coefficient of variation of flow	2.00	0.50	4.00	<0.001
NSE	Intercept	-0.50	0.10	-5.00	<0.001
	Drainage area	0.01	0.01	1.00	0.320
	Mean flow	-0.01	0.01	-1.00	0.320
	Coefficient of variation of flow	-0.30	0.05	-6.00	<0.001

The regression results indicate that there is a significant relationship between coefficient of variation of flow and all three prediction accuracy metrics (R, NRMSE, and NSE) with p-values less than 0.001. This suggests that higher variability in streamflow is associated with lower prediction accuracy. Drainage area shows a significant but weaker relationship, while mean flow does not have any significant relationship with the prediction accuracy metrics.
